# Supplementary material for: Associations of Cognitive Function with Serum Magnesium and Phosphate in Hemodialysis Patients: A Cross-Sectional Analysis of the Osaka Dialysis Complication Study (ODCS)
Source: Nutrients. 2024 Nov 3;16(21):3776. doi: 10.3390/nu16213776 (PMC11547714; doi:10.3390/nu16213776)
Supplement: Supplementary file 1 [file nutrients-16-03776-s001.zip › nutrients-3274331-supplementary.pdf]

Supplementary materials

Table S1. Dialysate compositions and the numbers of users

Dialysate	Na^+ mEq/L	K^+ mEq/L	Ca^{++} mEq/L	Mg^{++} mEq/L	Cl^- mEq/L	HCO_3^- mEq/L	CH_3COO^- mEq/L	$Citrate^{3+}$ mEq/L	Glucose mg/dL	Users N (%)
A	140	2.0	2.5	1.0	114.5	25	8	0	150	86 (5.1%)
B	138	2.0	2.5	1.0	110	28	8	0	100	249 (14.7%)
C	140	2.0	2.75	1.0	112.25	27.5	8	0	125	360 (21.2%)
D	140	2.0	3.0	1.0	110	30	8	0	100	735 (43.3%)
E	140	2.0	3.0	1.0	113	25	10	0	100	102 (6.0%)
F	140	2.0	3.0	1.0	113	25	10.2	0	100	66 (3.9%)
G	140	2.0	3.0	1.0	111	35	0	2.0	150	98 (5.8%)

The table gives seven different dialysate compositions used for patients in this study and the numbers (proportions) of patients. Note that no dialysate contained phosphate.

Table S2. Proportions of patients with low serum magnesium and low serum phosphate levels by 3MS quartile

Low serum levels	Q1 (N = 309)	Q2 (N = 299)	Q3 (N = 356)	Q4 (N = 243)	p-value	Missing, N (%)
Serum magnesium < 1.8 mg/dL	2 (0.7%)	1 (0.3%)	1 (0.3%)	1 (0.4%)	0.895	0 (0%)
Serum phosphate < 4.0 mg/dL	76 (24.6%)	44 (14.7%)	51 (14.3%)	39 (16.0%)	0.002	0 (0%)
Serum phosphate < 3.5 mg/dL	33 (10.7%)	18 (6.0%)	22 (6.2%)	18 (7.4%)	0.097	0 (0%)
Serum phosphate < 3.0 mg/dL	16 (5.2%)	6 (2.0%)	9 (2.5%)	6 (2.5%)	0.094	0 (0%)

The table gives numbers (percentages) of patients with lower levels of serum magnesium and phosphate by quartile of 3MS. P-values by χ^2 test. Reference range of serum magnesium is between 1.8 and 2.6 mg/dL. The recommended range of serum phosphate is between 3.5 and 6.0 mg/dL according to the 2013 version of the clinical practice guideline for the management of chronic kidney disease-mineral and bone disorder by the Japanese Society for Dialysis Therapy.

Abbreviations: Q, quartile; 3MS, Modified Mini-Mental State examination.

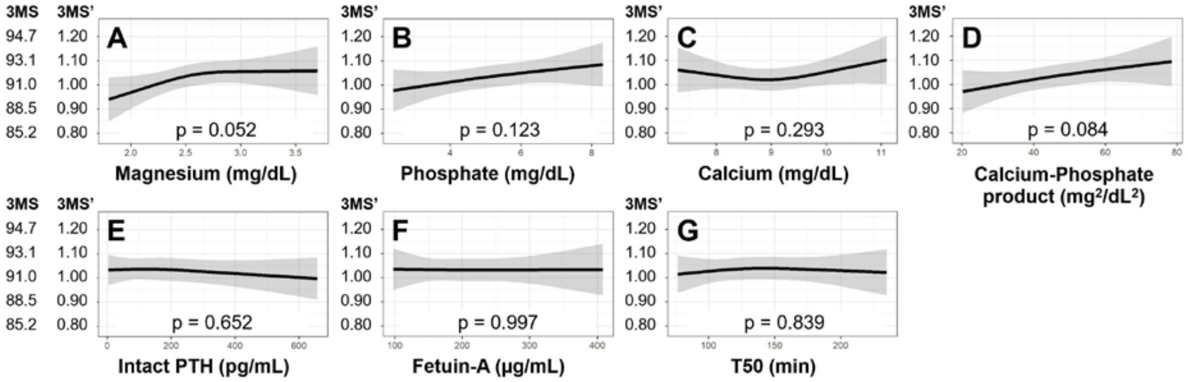

Figure S1. Associations of 3MS with seven MBD-related serum parameters by multivariable-adjusted nonlinear regression models (sensitivity analysis).

Associations of 3MS and MBD-related serum parameters were examined by multivariable-adjusted nonlinear regression analysis using restricted cubic spline function. Solid lines and shaded area are regression lines and 95% confidence intervals. 3MS was mathematically transformed before entering the model: 3MS' = 2 – Log10(101 − 3MS). Panels A through G show the results for serum magnesium, phosphate, calcium, calcium-phosphate product, intact PTH, magnesium, fetuin-A, and T50, respectively.

Abbreviations: 3MS, Modified Mini-Mental State examination; PTH, parathyroid hormone; T50, T50 calciprotein crystallization test.
